# Identification and Genome Sequencing of Novel Virulent Strains of *Xanthomonas oryzae* pv. *oryzae* Causing Rice Bacterial Blight in Zhejiang, China

**DOI:** 10.3390/pathogens13121083

**Published:** 2024-12-09

**Authors:** Weifang Liang, Yuhang Zhou, Zhongtian Xu, Yiyuan Li, Xinyu Chen, Chulang Yu, Fan Hou, Binfeng Dai, Liequan Zhong, Ji-An Bi, Liujie Xie, Chengqi Yan, Jianping Chen, Yong Yang

**Affiliations:** 1 College of Plant Protection, Yunnan Agricultural University, Kunming 650000, China; liangwf0102@163.com; 2 State Key Laboratory for Managing Biotic and Chemical Threats to the Quality and Safety of Agro-Products, Key Laboratory of Biotechnology in Plant Protection of MOA of China and Zhejiang Province, Institute of Virology and Biotechnology, Zhejiang Academy of Agricultural Science, Hangzhou 310000, China; 2301130077@nbu.edu.cn (Y.Z.); chenxinyu8262@gmail.com (X.C.); 3 State Key Laboratory for Managing Biotic and Chemical Threats to the Quality and Safety of Agro-Products, Key Laboratory of Biotechnology in Plant Protection of MOA of China and Zhejiang Province, Institute of Plant Virology, Ningbo University, Ningbo 315000, China; xuzhongtian@nbu.edu.cn (Z.X.); liyiyuan@nbu.edu.cn (Y.L.); yuchulang@nbu.edu.cn (C.Y.); 4 College of Plant Protection, Shenyang Agricultural University, Shenyang 110000, China; 5 Wuwangnong Seed Shareholding Co., Ltd., Hangzhou 310000, China; houfan1986@126.com; 6 Taizhou Agroecological Protection and Quality Safety Center, Taizhou 318000, China; lindabridget@163.com (B.D.); zhongliequan999@163.com (L.Z.); 7 Institute of Biotechnology, Ningbo Academy of Agricultural Sciences, Ningbo 315000, China; bihappy@foxmail.com (J.-A.B.); yanchengqi@163.com (C.Y.); 8 Taizhou Academy of Agricultural Sciences, Taizhou 318000, China; xieliujie@126.com

**Keywords:** avirulence gene, genome sequencing, TAL effector, *Xanthomonas oryzae* pv. *oryzae*

## Abstract

*Xanthomonas oryzae* pv. *oryzae* (*Xoo*) is the causative agent of rice bacterial blight (RBB), resulting in substantial harvest losses and posing a challenge to maintaining a stable global supply. In this study, *Xoo* strains isolated from Shaoxing, Quzhou, and Taizhou, where RBB occurred most frequently in Zhejiang Province in 2019, were selected as the subjects of research. Three isolated pathogenic bacteria of ZXooS (from Shaoxing), ZXooQ (from Quzhou), and ZXooT (from Taizhou) were all identified as novel *Xoo* strains. These novel strains demonstrate greater virulence compared to Zhe173, the previous epidemic *Xoo* strain from Zhejiang Province. Subsequent genomic sequencing and analysis revealed that there existed significant differences in the genome sequence, especially in effector genes corresponding to some known rice resistance (*R*) genes between the novel strains and Zhe173. The sequence alignment of avirulent genes (effector genes) indicated that nucleic and amino acid sequences of *AvrXa5*, *AvrXa7*, *AvrXa10*, and *AvrXa23* in the novel strains varied prominently from those in Zhe173. Interestingly, it seemed that only the genome of ZXooQ might contain the *AvrXa3* gene. In addition, the phylogenetic analysis of 61 *Xoo* strains revealed that the novel strains were situated in a distinct evolutionary clade separate from Zhe173. These results here suggest that the emergence of novel *Xoo* strains may lead to resistance loss of some *R* genes used in commercial rice varieties, potentially serving as one of the factors leading to RBB resurgence in Zhejiang Province, China.

## 1. Introduction

Rice bacterial blight (RBB) is the most devastating bacterial disease affecting rice worldwide, seriously endangering rice yield. It has been reported that RBB typically results in a 10–30% yield loss, and in severe cases, it can lead to a reduction of more than 50% in yield or even complete crop failure [1,2,3,4]. RBB was first discovered in 1884 in rice paddies in the Fukuoka region of the island of Kyushu, Japan. Subsequently, RBB was also found in several Asian, African, and Australian countries. Since the 1960s, the epidemic of RBB has spread worldwide. From the 1980s to the beginning of this century, with the implementation of a series of preventive measures such as the cultivation and utilization of a large number of commercial rice resistance varieties, RBB has gradually been effectively controlled. However, since 2014, RBB has exhibited a resurgence in the Yangtze River Basin, particularly in Zhejiang Province, with a growing frequency and expanding geographical coverage. There is still a lack of comprehensive research on the cause of RBB re-emergence in Zhejiang Province, which has sparked numerous debates. Some studies even suggest that the epidemic may be caused by new pathogens, such as *Pantoea ananatis* [5].

RBB is a vascular disease caused by the Gram-negative *Xanthomonas oryzae* pv. *oryzae* (*Xoo*), which invades through the water holes or wounds of rice leaf, and then enters the xylem duct to grow rapidly there. The infestation of *Xoo* has caused the spread of RBB worldwide. *Xoo* is a rapidly evolving pathogen [6], and the selection of rice resistance varieties may also facilitate the shift in races or result in the emergence of new races of *Xoo*. To date, over *Xoo* 30 races have been isolated and identified all over the world [7,8]. Different identification systems are used to distinguish physiological races in different countries since studies have shown that *Xoo* differentiation in different countries has its own characteristics. For example, the system of the International Rice Research Institute contains at least 10 Philippines *Xoo* races of Race1 to Race10: R1 (PXO61), R2 (PXO86), R3 (PXO79), R4 (PXO71), R5 (PXO112), R6 (PXO99), R7 (PXO145), R8 (PXO280), R9 (PXO330), and R10 (PXO341). The system of Japan contains at least 9 Japanese *Xoo* races of IA, IB, II, IIIA, IIIB, IV, V, VI, and VII, such as Race I (T7174), Race II (T7147), Race III (T7133), Race IV (H75373), Race V (H75304), and Race VII (H8584). The system of China contains at least 9 Chinese *Xoo* races of Race1 to Race9, such as R1 (YN18), R2 (YN1), R3 (GD414), R4 (Zhe173), R5 (ScYc-b), R6 (YN7), R7 (YN11), R8 (FuJ), and R9 (YN24). Zhe173 is a former strongly virulent *Xoo* strain of Chinese Race4, dominant in the Yangtze River valley in China [9]. In Zhejiang Province, Zhe173 has long been regarded as a representative *Xoo* strain and is widely used in rice cultivar resistance identification experiments [10,11,12].

Under the dual pressures of natural and artificial selection, *Xoo* is prone to mutation, resulting in the ongoing emergence of RBB [13,14]. So, it is necessary to reappraise and study *Xoo* variation at intervals in various regions to advance RBB research and implement effective prevention measures. However, the data for *Xoo* in Zhejiang Province have hardly been updated for a long time. Furthermore, there is still a lack of relevant research on the possibility that *Xoo* variation is one of the causes of RBB re-emergence in Zhejiang Province, China. Therefore, investigating the occurrence of RBB and analyzing the genomic variation in the *Xoo* strain will be beneficial for the effective control of RBB and the development of disease-resistant breeding in rice production.

In the present study, we conducted isolation, identification, and genomic sequencing and analysis on three *Xoo* strains from three areas with the most severe RBB occurrence in Zhejiang Province, China, including Shaoxing, Quzhou, and Taizhou. The results here revealed significant variations in the genome sequence, particularly in effector genes corresponding to some known resistance (*R*) genes between the three novel and former dominant *Xoo* strains in Zhejiang Province, which is suggested as one of the crucial reasons for the RBB re-epidemic in this province. Additionally, this study provides an important basis for subsequent research on the coevolution between *Xoo* effector and rice *R* genes in this province and future identification of novel rice *R* genes, which is crucial for efficient RBB prevention and management in Zhejiang Province, China.

## 2. Materials and Methods

### 2.1. Isolation and Identification of Xoo

The collected infected leaves were washed with sterile water to remove dust and dirt, soaked in 75% disinfecting alcohol for 1 min, soaked in 30% sodium hypochlorite for 5 min, and rinsed with sterile water three times, and the diseased leaves were then cut into pieces using sterile scissors, placed on an inclined PSA medium, cultured at 28 °C, and checked regularly. A single colony was selected and a pure culture was obtained using the continuous streaking method. The individual colonies were selected and transferred into liquid PSA medium and then incubated at 220 rpm for 48 h at 28 °C in a shaker (OD_600_ = 0.6~0.8). Then, DNA was extracted from the shaken bacterial solution using a Trelief^®^ Bacteria Genomic DNA Kit (TSINGKE TSP701-50). 16S rRNA gene primers and gyrB gene primers (Table S7) [5,15] were used to amplify bacterial DNA via PCR, and the resulting PCR products were sent to sequencing companies for analysis. The obtained sequences were BLASTed on the NCBI website.

### 2.2. Pathogen Inoculation and Disease Evaluation

The bacterial blight strain Zhe173 and the novel *Xoo* strains ZXooS (*Xoo* strain from Shaoxing), ZXooQ (*Xoo* strain from Quzhou), and ZXooT (*Xoo* strain from Taizhou) were cultured at 28 °C for 48 h, and the density of *Xoo* was adjusted to 10^8^ CFU/mL. Then, Yongyou19 (the main commercial rice cultivar in Zhejiang Province) rice plants were inoculated with *Xoo* in the tillering stage using the leaf-clipping method. After 21 days of inoculation, the length of the lesions was measured from the incision site to the withered area on the leaves, and the occurrence of lesions on rice leaves was scanned and recorded.

### 2.3. Genome Sequencing and Assembly

Genomic DNA from the *Xoo* samples (ZXooS, ZXooQ, ZXooT) were extracted using SDS [16], followed by agarose gel electrophoresis to assess the purity and integrity of the DNA, and quantified using Qubit. PacBio Sequel II and Illumina NovaSeq 6000 sequencing was performed by Hangzhou Lianchuan Biologics. After the sequencing was completed, the raw data were filtered to obtain high-quality sequencing reads. Based on a variety of quality control data, the genome was assembled using SMRT Link v5.0.1 software (https://www.pacb.com/support/software-downloads/, accessed on 26 May 2020.) [17,18]. Next, Arrow software was utilized to optimize the assembly results and rectify any areas with assembly errors. The optimized assembly results were compared and analyzed. The second-generation data were used for correction, and the chromosomes and plasmid sequences were screened. The chromosome sequences were assembled into a circular genome (or a linear genome if it was a linear genome sequence), resulting in the final 0gap completion graph sequence. GeneMarkS (Version 4.17) (http://topaz.gatech.edu/GeneMark/, accessed on 28 May 2020) software [19] was utilized to predict the coding genes of the newly sequenced genomes. tRNA was predicted using tRNAscan-SE software (Version 1.3.1) [20]. rRNA prediction was performed using rRNAmmer software (Version 1.2) [21]. First, the Rfam database [22,23] was annotated, and then the final sRNA was determined using the cmsearch program (Version 1.1rc4) (parameter default). In addition, EffectiveT3 software [24] (Version 1.0.1) was used to predict T3SS effector proteins.

### 2.4. Comparative Genomics Analysis and Phylogenomic Analysis

For structural comparison, complete genomes were aligned using progressive Mauve with default settings [25]. For phylogenomic analysis, the genome sequences of 57 *Xoo* strains were obtained from the NCBI database (https://www.ncbi.nlm.nih.gov/genome/browse/#!/prokaryotes/529/, accessed on 10 October 2023), and OrthoFinder 2.2.7 was used to construct a phylogenetic tree based on the results of multiple sequence alignment (MSA) using the maximum likelihood method [26]. We used RAxML-NG to infer the evolutionary tree, and Figtree was used to visualize the phylogenetic tree. Different clades were color-coded.

## 3. Results

### 3.1. Isolation and Identification of Pathogenic Bacteria

**Figure 1:**
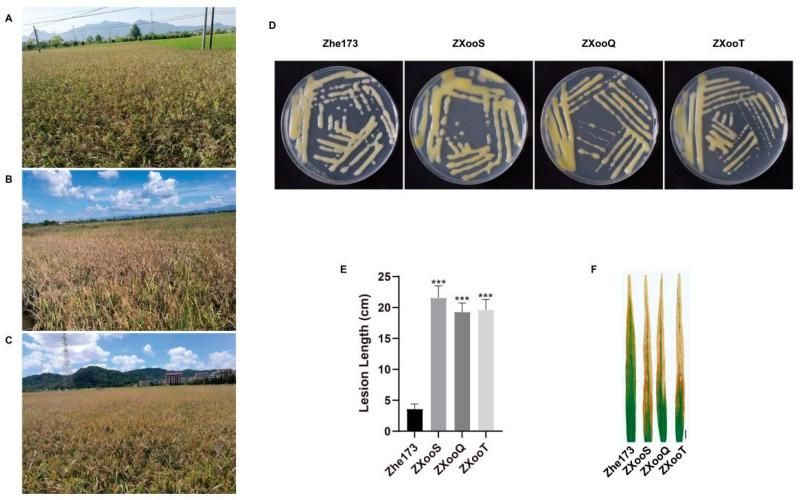
Isolation and identification of pathogenic bacteria. (**A**–**C**): Occurrence of RBB in Shaoxing, Quzhou, and Taizhou rice regions of Zhejiang Province. (**D**): Colony morphologies of *Xoo* strains from left to right are as follows: Zhe173, Shaoxing (ZXooS), Quzhou (ZXooQ), and Taizhou (ZXooT). (**E**): Lesion length of Yongyou19 after inoculation with Zhe173, ZXooS, ZXooQ, and ZXooT. Statistical analysis was performed by *t*-test (*** *p* < 0.001). (**F**): Disease phenotypes of Yongyou19 after inoculation with Zhe173, ZXooS, ZXooQ, and ZXooT. Bar = 1 cm.

We collected diseased rice samples from three areas with the most severe RBB occurrence of Quzhou, Shaoxing, and Taizhou in Zhejiang Province, China (Figure 1A–C). The pathogenic bacteria isolated from diseased rice leaves formed light yellow circular colonies with smooth margins on the culture medium and grew well within 48–72 h at 28 °C (Figure 1D). The sequences of DNA fragments amplified with 16S rRNA primers from the genomes of these three bacteria shared about 98% similarity with that of *Xoo* type strain PXO61 (Supplementary Table S1). The amplification products with *gyrB* primers also showed the highest homology to the PXO61 *gyrB* gene sequence with a similarity of 97–98% (Supplementary Table S1), which supported the above results. To test the virulence, the isolated bacteria were used to inoculate Yongyou19. It was found that the three *Xoo* strains ZXooS, ZXooQ, and ZXooT were much more virulent than the previous strain Zhe173, causing longer lesion length on rice leaves (Figure 1E,F). In addition, the pathogenic bacteria re-isolated from the inoculated rice leaves were also identified as the same *Xoo* strains. Taken together, the complete identification process according to Koch’s postulates confirms that the pathogen causing RBB resurgence in Zhejiang Province is still *Xoo* rather than other types of pathogens.

### 3.2. ZXooS, ZXooQ, and ZXooT Genome Sequencing Reveals Their Unique Features

**Figure 2:**
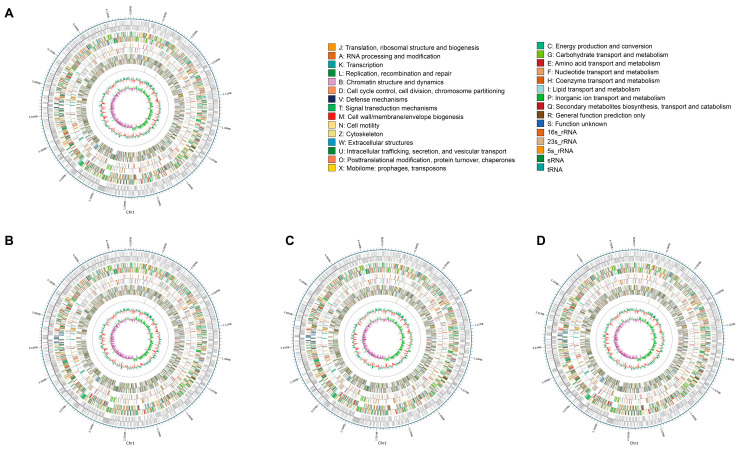
Genome features of *Xanthomonas oryzae* pv. *oryzae* Zhe173 (**A**), ZXooS (**B**), ZXooQ (**C**), and ZXooT (**D**). The outermost circle represents the genome sequence position coordinates. Moving from the outside to the inside, it includes the coding genes, gene function annotation results, ncRNA, and genomic GC content. The inner red part indicates that the GC content of this region is lower than the average GC content of the whole genome, while the outer green part indicates the opposite. The higher the peak value, the greater the difference from the average GC content and the genomic GC skew value. The inner pink part indicates that the content of G is lower than the content of C in this region, while the outer green part indicates the opposite.

As shown in Figure 2 and Table 1, genomic sequencing and assembly showed that each of the four genomes comprised one individual circular chromosome. The genomes of the three newly identified strains were all larger than that of Zhe173, with 4924181, 4925078, 4931109, and 4914970 base numbers for ZXooS, ZXooQ, ZXooT, and Zhe173, respectively. Moreover, the sequenced coding sequences in the genomes of the three strains (4942, 4940, and 4950 for ZXooS, ZXooQ, and ZXooT, respectively) all exceeded those of Zhe173 (4926). Each of the four genomes contained the same number of six ribosomal RNA genes; ZXooS had the same number of 52 transfer RNA genes as Zhe173, while both ZXooQ and ZXooT had one more than Zhe173. According to the annotation of Clusters of Orthologous Groups (COG), the Zhe173 genome included 3940 predicted genes encoding proteins, slightly more than ZXooS (3934) and ZXooQ (3938), and slightly less than ZXooT (3941). There were 3704 predicted genes in the Zhe173 genome on the basis of the database of Kyoto Encyclopedia of Genes and Genomes (KEGG), and the number of such genes in ZXooS (3699) was less than in Zhe173, while this number in ZXooQ (3711) and ZXooT (3708) was more than in Zhe173. There were 319, 320, and 318 predicted secreted proteins in ZXooS, ZXooQ, and ZXooT, respectively, all exceeding the 310 ones in Zhe173, and among such secreted proteins, the number of transcription activator-like effectors (TALEs) was the same in ZXooT (16) as in Zhe173 (16), while there was one more in both ZXooS (17) and ZXooQ (17). In brief, the results here reveal that there exist significant differences in the genome sequence between the newly identified strains and Zhe173; especially, the number of some of their predicted sequence functional units, such as the coding sequence and secreted proteins, all exceed that of Zhe173, which indicates that they may have evolved a more complex genetic system than Zhe173.

### 3.3. ZXooS, ZXooQ, and ZXooT Were Located on the Same Branch of the Evolutionary Tree and Were Different from Zhe173

**Figure 3:**
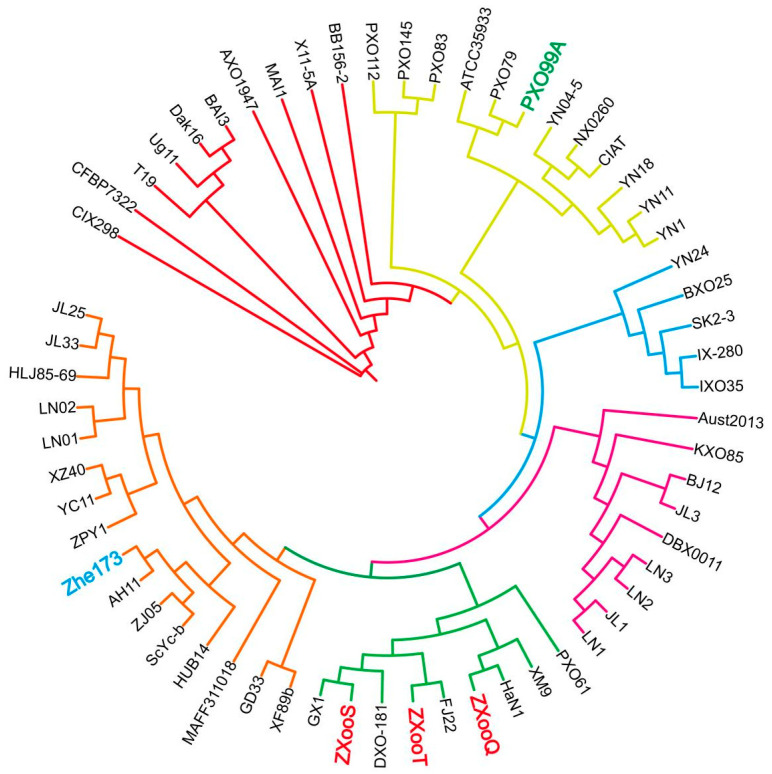
Phylogenetic tree of 61 *Xoo* strains from various regions. Red represents the African strains; yellow represents the South Asian strains (India, Nepal), Southeast Asian strains (Philippines), and East Asian strain (Yunnan, China); blue represents the strains from India, Thailand, and China; purple represents East Asian strains from North China and Korea; green represents South China strains; and orange represents other Chinese and Japanese strains.

We conducted the phylogenetic tree analysis using the protein sequences of ZXooS, ZXooQ, ZXooT, Zhe173, and 57 other *Xoo* strains/isolates available (Table S2), which were mainly collected from Asia, Africa, South America, and Oceania. The 61 strains could be divided into six distinct clades, one of which was exclusively composed of African strains, while the other five mostly consisted of Asian strains (Figure 3), which suggests that the strains from Africa and Asia have already diverged in early stages. In the phylogenetic tree, the three newly identified Zhejiang strains of ZXooS, ZXooQ, and ZXooT all belonged to the same clade as the strains from Hainan (HaN1), Fujian (FJ22), Guangxi (GX1), Taiwan (XM9), and India (DXO181). Meanwhile, the previous Zhejiang strains Zhe173, ZPY1, and ZJ05 were located in another clade, which also included strains from Jilin (such as JL25), Liaoning (such as LN01), Hubei (such as HUB14), Sichuan (such as ScYc-b), and Anhui (AH11). These results above also support the inference that there are significant differences between the newly identified strains and the previous epidemic strain Zhe173.

### 3.4. Novel TALEs Encoded by the ZXooS, ZXooQ, and ZXooT Genome

*Xoo* attacks host plants primarily by secreting different effector proteins through the type III secretion system (T3SS), among which TALEs are the most special effector family usually binding to and activating the host susceptibility gene promoters. TALEs contain multiple 33–35-amino-acid-long sequence repeats, but differ in number and arrangement. The main variation between each repeat unit exists in the 12th and 13th amino acids. The combination of these two highly variable amino acids is called repeat variable di-residues (RVDs), which determine the recognition specificity of promoter motifs of host genes [27,28]. The predicted Zhe173 TALEs contain variable RVD sequences with repeats ranging from 13 to 27 (Supplementary Table S3). These repeats range from 13 to 27 in ZXooS (Supplementary Table S4), from 7.5 to 29 in ZXooQ (Supplementary Table S5), and from 13 to 26.5 in ZXooS (Supplementary Table S6). At the same time, more than half of the effectors’ RVD sequences of the novel strains differ from that of Zhe173 (Supplementary Tables S3–S6).

**Figure 4:**
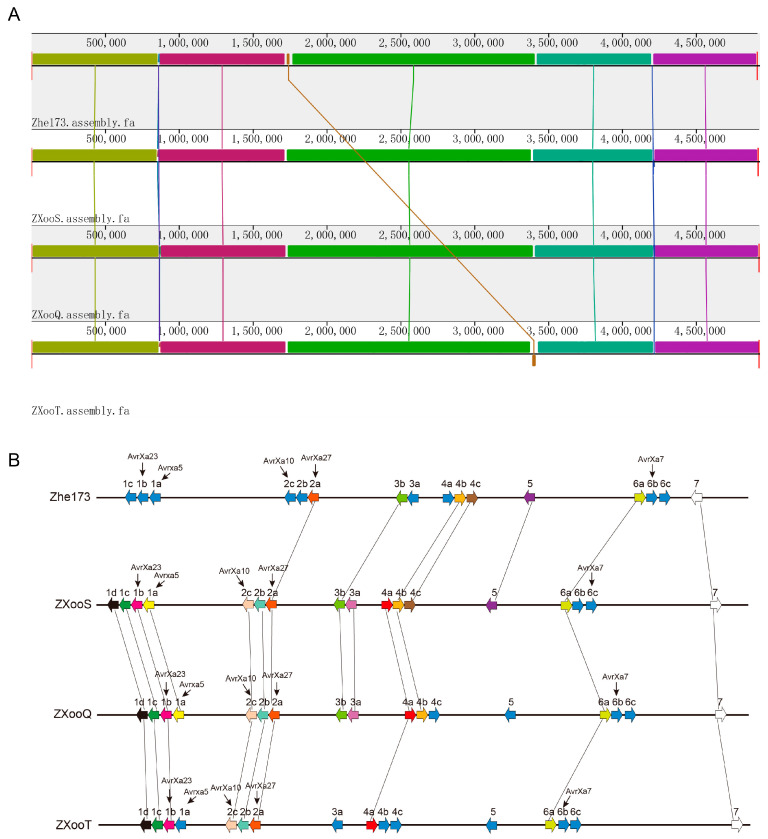
Comparison of whole genome and TALEs in *Xoo* strains. (**A**) Progressive Mauve alignment chromosomes of *Xoo* strains. The ruler indicates the distance from the annotated origin in base pairs. (**B**) The Tal genes of *Xoo* strains. Gene orientations are indicated by arrows. The identical effectors with the same RVD sequence are labeled with the same color and connected by lines. The effectors are identified based on genome sequences and are indicated in blue without lines.

Collinearity analysis showed that Zhe173 and the novel strains had distinct structural variations at two positions (Figure 4A and Figure S12). This indicated that there were differences between Zhe173 and the newly identified strains. Additionally, the numbers of *TAL* gene clusters are diversified between the novel strains and Zhe173 (Figure 4). There are seven TAL loci encoding 16 effectors in the Zhe173 genome, seven TAL loci for 17 effectors in ZXooS, seven for 17 in ZXooQ, and seven for 16 in ZXooT. And the sequences of AvrXa23, Avrxa5, AvrXa10, and AvrXa7 in the novel strains differed from those in Zhe173 (Figure 4). This variation may be related to pathogen-specific differences caused by the use of different resistance genes. These results suggest that the novel strain may have acquired new virulence.

### 3.5. The Avirulent Genes in ZXooS, ZXooQ, and ZXooT Were Mutated Compared to Zhe173

**Figure 5:**
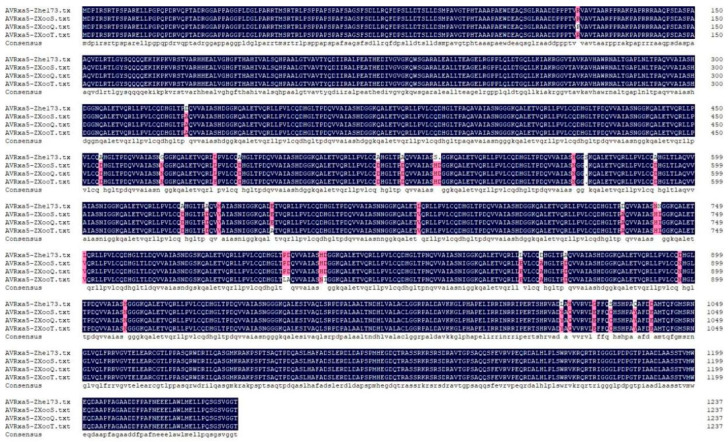
Comparison results of the *Avrxa5* gene amino acid sequence in Zhe173, ZXooS, ZXooQ, and ZXooT strains.

In the rice genome, the *R* genes against *Xoo* are named *Xa* genes, and the effector genes in the *Xoo* genome that *Xa* genes identify to trigger rice’s resistance response are traditionally called avirulent genes. Here, we used DNAMAN software to compare the nucleotide and amino acid sequences of *AvrXa3*, *Avrxa5*, *AvrXa7*, *AvrXa10*, *AvrXa23*, and *AvrXa27* in the genomes of Zhe173, ZXooS, ZXooQ, and ZXooT. In the comparison results of *Avrxa5* (GenBank accession number FJ593881.1), there were 37 amino acid differences (0.81%) and 0.52% nucleotide differences between Zhe173 and the novel strains (Figure 5, Supplementary Figure S3). Regarding *AvrXa7* (GenBank accession number AY626404.1), the amino acid comparison revealed a difference rate of 3.55% (Supplementary Figure S4). In the nucleotide comparison, the difference rate was 4.4% (Supplementary Figure S5). In the comparison results of *AvrXa10* (GenBank accession number U50552.1), there were 74 amino acid differences (1.92%) and a 1.14% nucleotide difference rate between Zhe173 and novel strains (Supplementary Figures S6 and S7). In the comparison results of *AvrXa23* (GenBank accession number GU732172.1), there were 39 amino acid differences (1.08%) and a 0.83% nucleotide difference rate between Zhe173 and novel strains (Supplementary Figures S8 and S9). On the other hand, when comparing *AvrXa27* (GenBank accession number AY986494.1), it was found that the protein sequence of AvrXa27 was identical in all four strains and differed by only two bases in the comparison of nucleotides (Supplementary Figures S10 and S11). In addition, by using *AvrXa3* (GenBank accession number AY129298.1) as a reference, it was found that only the genome data of ZXooQ contained the *AvrXa3* gene. In the ZXooQ genome, AvrXa3 showed 80.20% homology with the reference protein AvrXa3 when comparing their amino acid sequences and showed 83.67% homology with the reference gene *AvrXa3* when comparing their nucleotide sequences (Supplementary Figures S1 and S2). Nevertheless, there are no genes with strong resemblance to the *AvrXa3* gene in the genomes of ZXooS, ZXooT, or Zhe173. Genes in the ZXooS, ZXooT, and Zhe173 genome that are homologous to *AvrXa3* only exhibit 67–73% identity. For example, in the Zhe173 genome, the AvrXa3 protein showed only 67.49% homology with the reference protein AvrXa3 when comparing their amino acid sequences. Based on the analysis above, we speculate that due to the long application of the *Xa3* gene and other reasons, AvrXa3 has undergone multiple mutation and produced a large number of copy forms to long-term-cope with the resistance of various rice varieties.

## 4. Discussion

In recent years, RBB has reemerged and broke out in Zhejiang Province, the primary rice-producing region of China. In this study, three *Xoo* strains, ZXooS, ZXooQ, and ZXooT, were isolated from the leaves of three areas heavily affected by RBB in Quzhou, Shaoxing, and Taizhou, Zhejiang Province. Whole-genome sequencing and analysis revealed significant changes in the genomes of three newly isolated strains, ZXooS, ZXooQ, and ZXooT, compared to the epidemic strain Zhe173 in Zhejiang Province. There were significant differences in genome size, structure, and the number of encoded proteins. Especially, the identified effector genes of many RBB resistance genes have also changed significantly. In addition, the results of inoculation experiments on the main rice variety Yongyou19 in Zhejiang Province also showed that ZXooS, ZXooQ, and ZXooT were much more pathogenic than Zhe173. All of these results indicate that ZXooS, ZXooQ, and ZXooT are three novel *Xoo* strains different from Zhe173. The genomic resources of these strains provide information for discovering novel virulence effectors and comprehending the virulence mechanisms of TALEs in rice.

The pathogenicity of pathogens and the resistance of their hosts are a pair of mutually evolving relationships. When pathogens invade plants, the plants recognize pathogen-associated molecular patterns (PAMPs) and trigger the early immune response known as PAMP-triggered immunity (PTI) [29,30]. In response to this infection, plants have evolved a more efficient secondary resistance response called effector-triggered immunity (ETI). Resistance (R) proteins are used to recognize the effectors released by pathogens, thereby activating various downstream resistance pathways [31]. The R protein of rice, which responds to RBB, is commonly known as the XA protein. The effector protein in *Xoo* is referred to as the Avr protein, which is typically classified as transcription activator-like effectors (TALEs) and non-TAL effectors. At present, most of the Avr proteins corresponding to the XA proteins are of the TALE type [32]. TALE monomers recognize specific bases in the *Xa* gene promoter through RVDs to activate *Xa* gene expression, thereby initiating the resistance response in rice. Therefore, the RVD region of the TALE-type Avr protein is an important recognition site for the rice XA protein to trigger resistance. It is also a crucial region for *Xoo* to mutate in order to evade the resistance induced by the *Xa* gene.

The Xa-Avr system is an extremely effective tool for controlling and preventing RBB. To date, at least 46 *Xa/xa* genes have been identified in rice [33]. Some of these important genes, such as *Xa3*/*Xa26* [34,35], *Xa4* [36,37], *xa5* [38,39], *Xa7* [40,41], *Xa10* [42,43], *xa13* [44,45], *Xa21* [46], *Xa23* [47], and *Xa27* [48], have been successfully isolated using a map-based cloning strategy. Many *Xa*/*xa* genes, including *Xa3*/*26*, *Xa4*, *xa5*, *Xa7*, *xa13*, *Xa21*, and *Xa23*, have been utilized in rice RBB resistance breeding [47,49,50,51,52,53,54]. Rice varieties carrying genes *Xa3*/*26*, *Xa4*, *xa5*, *Xa7*, and *Xa23* are widely planted in rice-growing regions in China. The utilization of these *Xa*/*xa* genes played a significant role in effectively controlling RBB in Zhejiang Province after the 1980s.

However, under long-term selective pressure from a single or a few *Xa*/*xa* genes, *Xoo* are prone to mutation, leading to the emergence of new strains, thereby losing the resistance of the existing *Xa*/*xa* genes, particularly due to mutations in the *Avr* gene. For example, *Xa23* with a high resistance has been widely used after its identification in the 2000s for controlling RBB in the middle and lower region of the Yangtze River of China [47]. Recently, it has been reported that a novel *Xoo* strain, AH28, from Anhui Province includes an *avrXa23* homolog, *tal7b,* with deletion of the 4th RVD and base mutations in the 5th/8th RVDs in its sequence. These alterations render the Tal7b protein unable to bind to the effector binding element (EBE) at the *Xa23* promoter, thereby preventing the activation of *Xa23* expression and consequently resulting in the loss of *Xa23*-induced RBB resistance [55]. Researchers isolated the *Xoo* strain LA20 from the rice variety Quanyou166 and compared it with the traditional YC11 strain from the Yangtze River. Significant differences were observed in the number and sequence of the Tal gene. This may be the primary reason why LA20 can overcome resistance mediated by *xa5*, *Xa7*, and *xa13*, leading to the re-emergence of bacterial blight in the Yangtze River region [14]. Additionally, whole-genome sequencing revealed that Liaoning strain LN4 possesses two major TALEs (Tal7 and Tal6c), which activate *OsSWEET13_kit_* and *OsSWEET14*. And this hypervirulent strain, LN4, is compatible in rice varieties carrying *Xa3*, *Xa4*, *xa13,* and *xa25* resistance genes. Once LN4 becomes prevalent, rice accessions containing the resistant genes *Xa3*, *Xa4*, *xa13*, and *xa25* will lose their resistance, leading to an outbreak of RBB [56]. Therefore, we propose that the outbreak of bacterial blight in rice in Zhejiang Province may also be related to the variation in the *Xoo* strain. In the present study, all of the three novel strains ZXooS, ZXooQ, and ZXooT are significantly different in *AvrXa* genes. There are four mutations in the 6th (NN), 8th (HD), 9th (N*), and 14th (HI) RVDs in the Avrxa5 protein of ZXooS and ZXooQ, and seven in the 6th (NN), 8th (HD), 9th (NG), 14th (HI), 15th (N*), 16th (NI), and 18th (NG) RVDs in that of ZXooT. As for AvrXa7 protein, the ZXooS strain has three mutations in the 4th (HD), 5th (NI), and 24th (N*) RVDs. And ZXooQ strain differences occurred at the 16th RVD, where “NS” in ZXooQ is “HD” in Zhe173. And AvrXa7 in the ZXooT strain, in which the 5th (NS), 11th (NS), 16th (NN), and 25th (NS) RVDs are altered. Three novel strains share the same sequence of the *AvrXa10* gene and show three differences from Zhe173 in the 12th, 14th, and 17th RVD of the AvrXa10 protein. For *AvrXa23*, the effector gene corresponding to the most widely used *Xa* gene in Zhejiang Province, there are five different RVDs between the novel strains and Zhe173: the 5th (NI), 13th (HD), 17th (NI), 18th (NI), and 24th (NG) RVDs in the novel strains, and the 5th (NS), 13th (NG), 17th (NN), 18th (NQ), and 24th (HG) RVDs in the Zhe173 strain (Figure 4 and Supplementary Tables S3–S6). The RVD array of AvrXa27 in novel strains is identical to that of the Zhe173 strain.

Since the *Xa3* gene has been utilized earlier in rice for anti-pathogenic breeding and has a wide range of applications, it is speculated that the *AvrXa3* genes have undergone several variations to overcome the resistance of various anti-pathogenic genes, which has led to the appearance of multiple forms of the gene. In this study, many similar loci of the *AvrXa3* gene were found in the genome of Zhe173 and three novel strains, but their homology was much lower than that of other *AvrXa* genes, indicating significant changes in the *AvrXa3* gene long before Zhe173. Consequently, the novel strains differed significantly from Zhe173 in terms of AvrXa3. The results above provide evidence supporting the conclusion that *Xoo* is prone to mutation. We also speculate that the mutation of *Xoo* to evade the resistance of the existing *Xa* gene may be one of the significant underlying reasons for the re-epidemic of RBB in Zhejiang Province. However, the more detailed mechanism needs to be further studied.

In addition, since the *AvrXa* gene of *Xoo* and the *Xa* gene of rice interact and evolve together, studying the *Xoo* genome, particularly the effector genes, can help analyze the effectiveness of resistance to existing *Xa* genes and identify new *Xa* genes. Therefore, the results of this study provide an important foundation for screening, cloning, and utilizing new rice disease resistance genes against the new *Xoo* strain in Zhejiang province. This research holds great significance for the effective prevention and control of RBB in Zhejiang Province.

## 5. Conclusions

Three novel *Xoo* strains were isolated from an area severely affected by RBB in Zhejiang Province. These strains exhibited strong pathogenicity toward the mainly cultivated rice varieties, Yongyou 19. Furthermore, we provide the complete genome sequences of these novel strains. The variation in the number and sequences of TALEs, particularly the sequence variation in *Avrxa* genes in the novel strains compared to Zhe173, may be responsible for the resurgence of RBB.

The complete genome sequence of Chinese Xoo strain Zhe173 has been deposited at GenBank under the accession number CP174352, ZXooS has been deposited at GenBank under the accession number CP174348, ZXooQ has been deposited at GenBank under the accession number CP174347, and ZXooT has been deposited at GenBank under the accession number CP174346.

## Figures and Tables

**Table 1:** Comparative analysis of the genomic features of Chinese *Xoo* strains ZXooS, ZXooQ, and ZXooT with Zhe173.

Genome Features	Zhe173	ZXooS	ZXooQ	ZXooT
Genome size (bp)	4,914,970	4,924,181	4,925,078	4,931,109
GC content (%)	63.71	63.72	63.7	63.72
Predicted CDS number	4926	4942	4940	4950
Total gene length (bp)	4,149,747	4,156,503	4,159,302	4,165,614
Gene average length (bp)	842	841	842	842
Gene length/genome (%)	84.43	84.41	84.45	84.48
rRNA number	6	6	6	6
tRNA number	52	52	53	53
COG annotated gene number	3940	3934	3938	3941
KEGG annotated gene number	3704	3699	3711	3708
Total secreted proteins number	310	319	320	318
TALE number	16	17	17	16

## Data Availability

The original contributions presented in this study are included in the article/Supplementary Materials, and further inquiries can be directed to the corresponding author.

## Supplementary Materials

The following supporting information can be downloaded at https://www.mdpi.com/article/10.3390/pathogens13121083/s1. Figure S1: Comparison results of the *AvrXa3* gene amino acid sequence in ZXooQ strains; Figure S2: Comparison results of the *AvrXa3* gene nucleotide sequence in ZXooQ strains; Figure S3: Comparison results of the *Avrxa5* gene nucleotide sequence in Zhe173, ZXooS, ZXooQ, and ZXooT strains; Figure S4: Comparison results of the *AvrXa7* gene amino acid sequence in Zhe173, ZXooS, ZXooQ, and ZXooT strains; Figure S5: Comparison results of the *AvrXa7* gene nucleotide sequence in Zhe173, ZXooS, ZXooQ, and ZXooT strains; Figure S6: Comparison results of the *AvrXa10* gene amino acid sequence in Zhe173, ZXooS, ZXooQ, and ZXooT strains; Figure S7: Comparison results of the *AvrXa10* gene nucleotide sequence in Zhe173, ZXooS, ZXooQ, and ZXooT strains; Figure S8: Comparison results of the *AvrXa23* gene amino acid sequence in Zhe173, ZXooS, ZXooQ, and ZXooT strains; Figure S9: Comparison results of the *AvrXa23* gene nucleotide sequence in Zhe173, ZXooS, ZXooQ, and ZXooT strains; Figure S10: Comparison results of the *AvrXa27* gene amino acid sequence in Zhe173, ZXooS, ZXooQ, and ZXooT strains; Figure S11: Comparison results of the *AvrXa27* gene nucleotide sequence in Zhe173, ZXooS, ZXooQ, and ZXooT strains; Figure S12: Comparison of whole genome between Zhe173 and novel strains. Table S1: Sequence identities of the obtained isolates with PXO61; Table S2: *Xoo* strains/isolates mainly from Asia, Africa, South America, and Oceania; Table S3: Repeat variable di-residue (RVD) sequences of *Xoo* strain Zhe173, where an asterisk indicates that the second amino acid in the RVD is absent; Table S4: Repeat variable di-residue (RVD) sequences of *Xoo* strain ZXooS, where RVDs in red are different in Zhe173 orthologs; Table S5: Repeat variable di-residue (RVD) sequences of *Xoo* strain ZXooQ; Table S6: Repeat variable di-residue (RVD) sequences of *Xoo* strain ZXooT; Table S7: Primer used for identification of pathogenic bacteria.
