# Supplementary material for: Identification and Genome Sequencing of Novel Virulent Strains of Xanthomonas oryzae pv. oryzae Causing Rice Bacterial Blight in Zhejiang, China
Source: Pathogens. 2024 Dec 9;13(12):1083. doi: 10.3390/pathogens13121083 (PMC11728688; doi:10.3390/pathogens13121083)
Supplement: Supplementary file 1 [file pathogens-13-01083-s001.zip › pathogens-3278515-supplementary Figure S1-S12.pdf]

Identification and Genome sequencing of novel virulent strains of *Xanthomonas oryzae* pv. *oryzae* causing Rice Bacterial Blight in Zhejiang, China

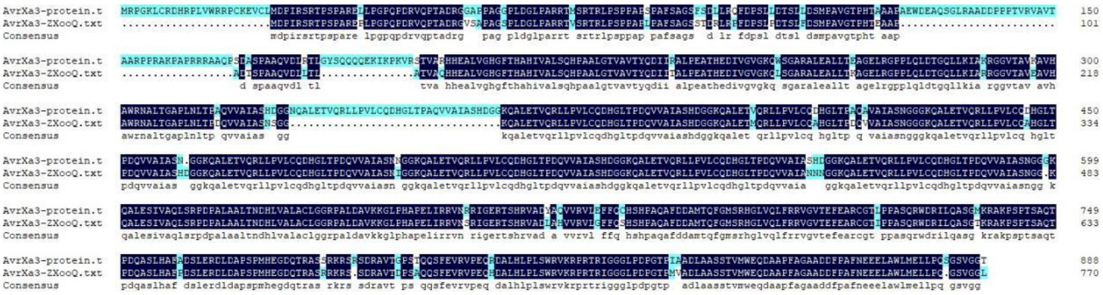

Figure S1. Comparison results of the AvrXa3 genes amino acid sequence in ZXooQ strains.

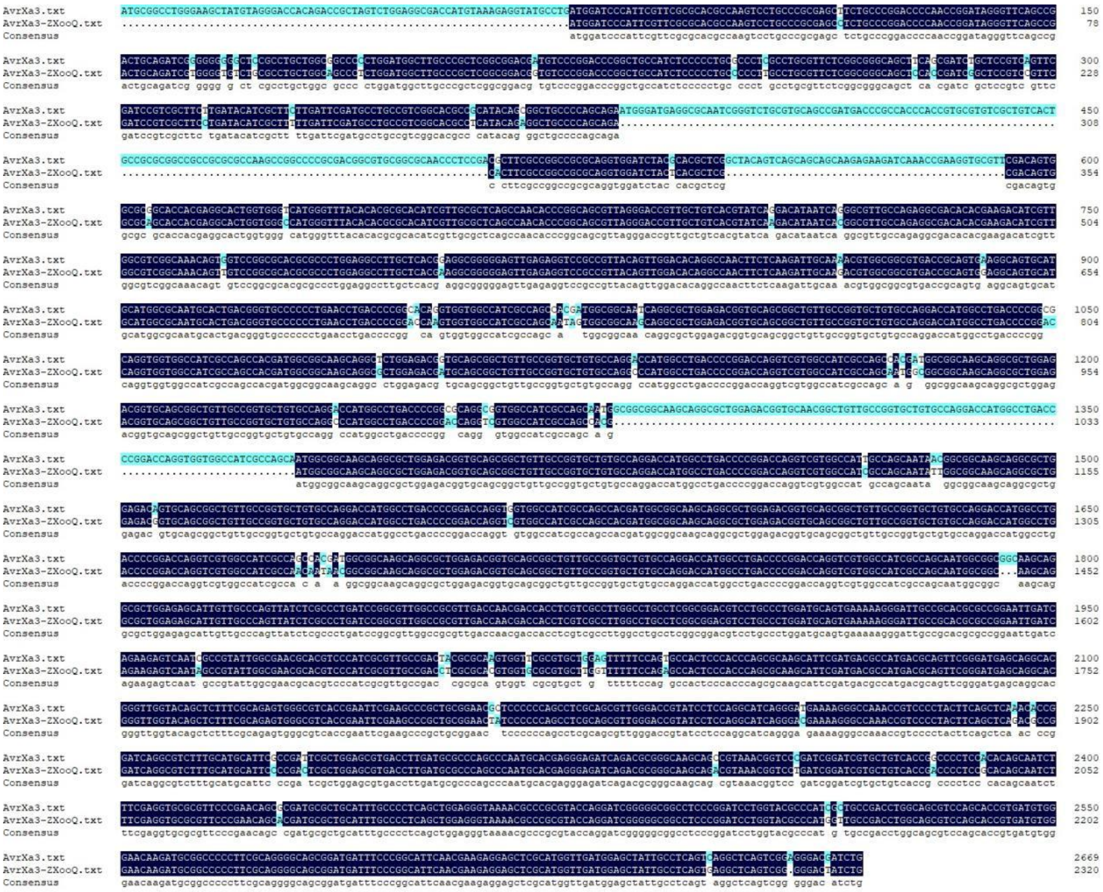

Figure S2. Comparison results of the *AvrXa3* genes nucleotide sequence in ZXooQ strains.

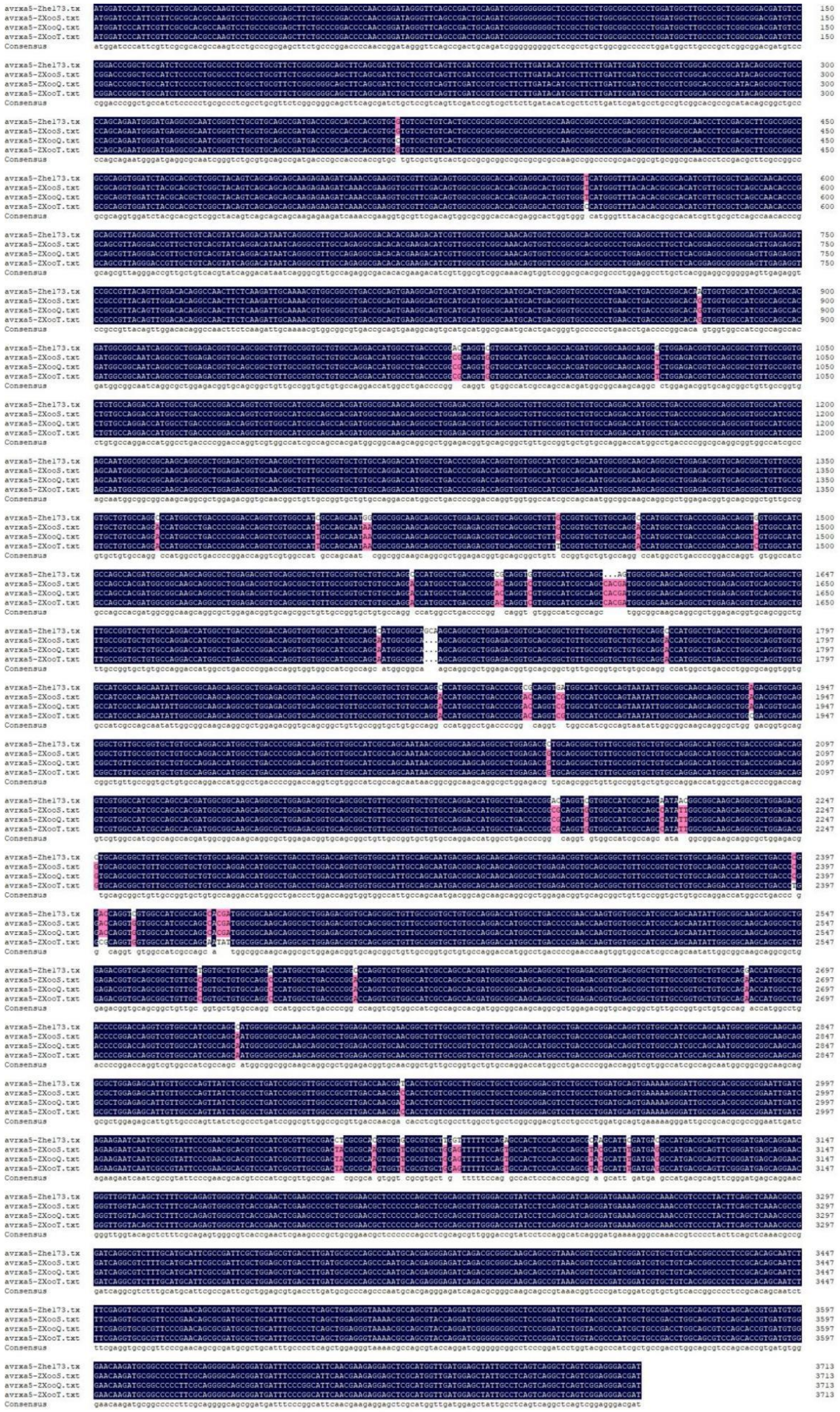

Figure S3. Comparison results of the *Avrxa5* genes nucleotide sequence in Zhe173, ZXooS, ZXooQ, and ZXooT strains.

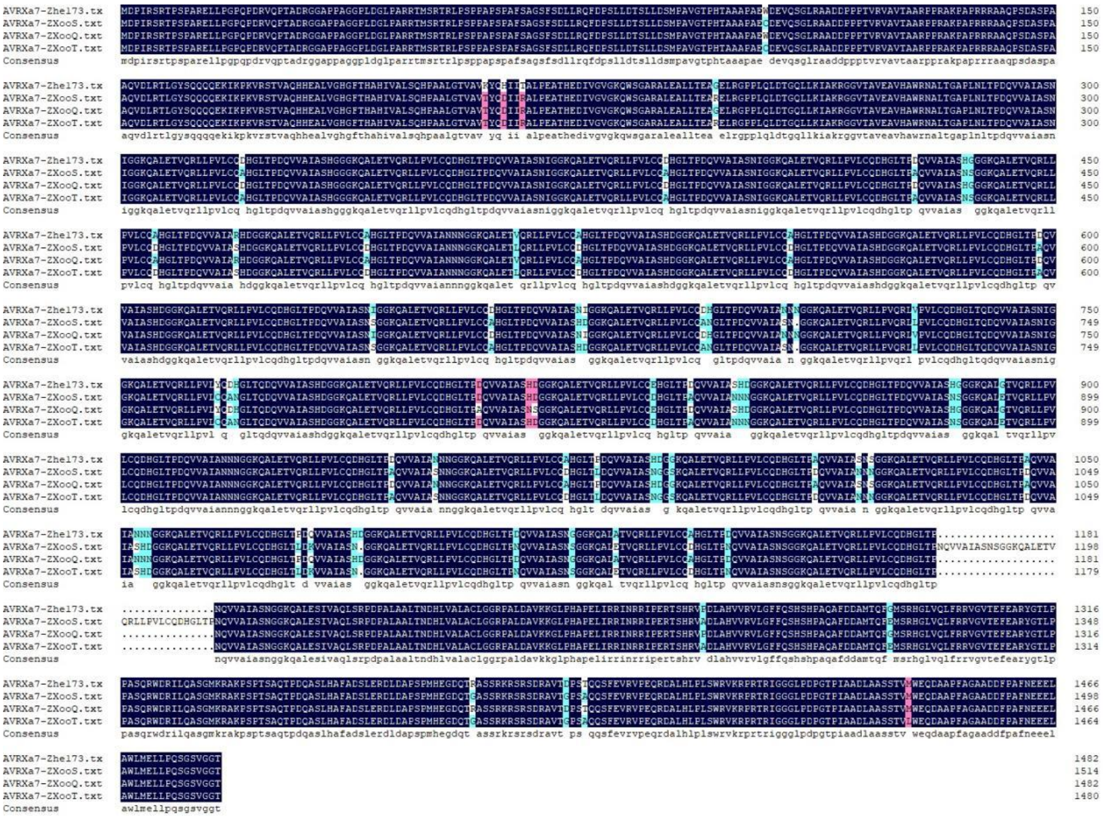

Figure S4. Comparison results of the *AvrXa7* genes amino acid sequence in Zhe173, ZXooS, ZXooQ, and ZXooT strains.

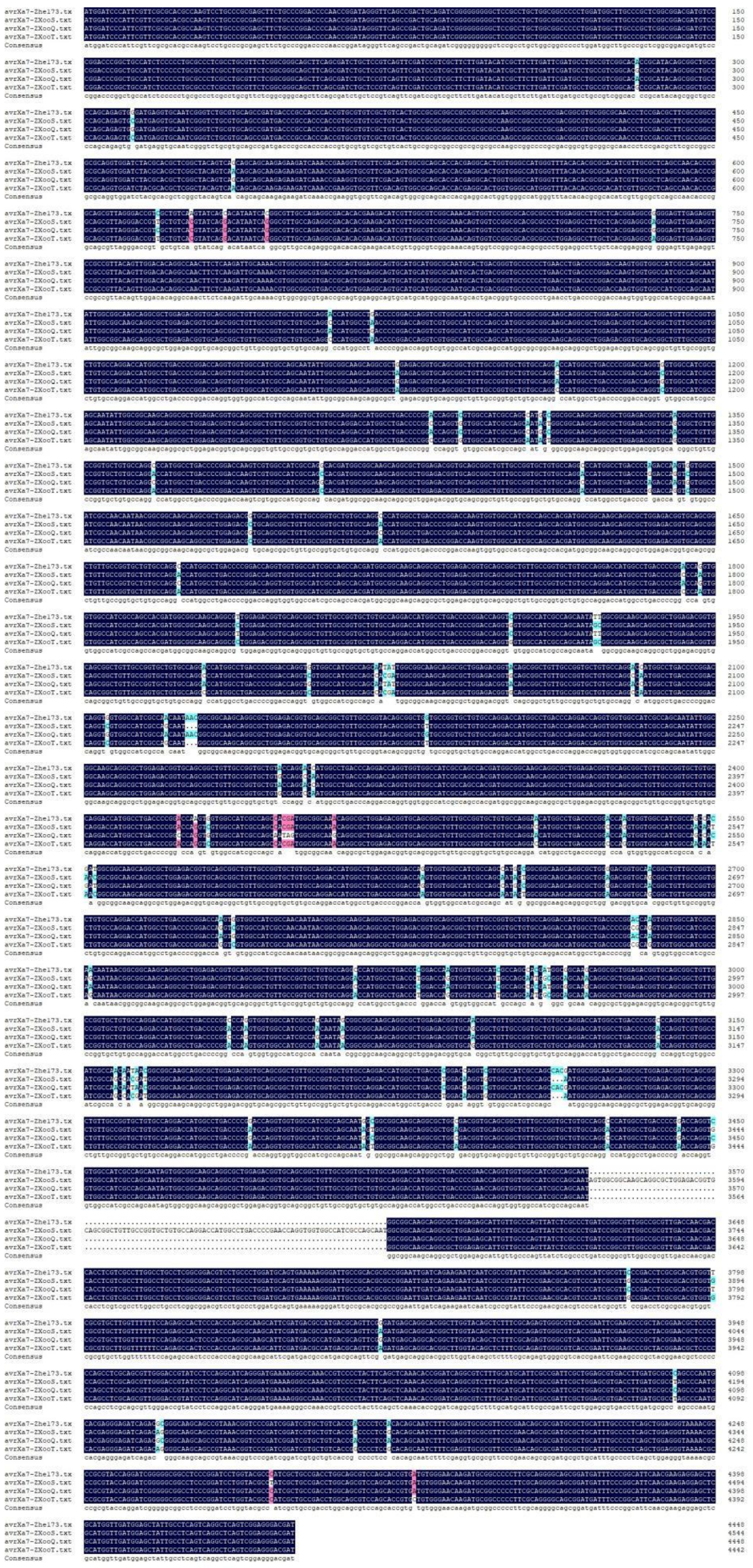

Figure S5. Comparison results of the *AvrXa7* genes nucleotide sequence in Zhe173, ZXooS, ZXooQ, and ZXooT strains.

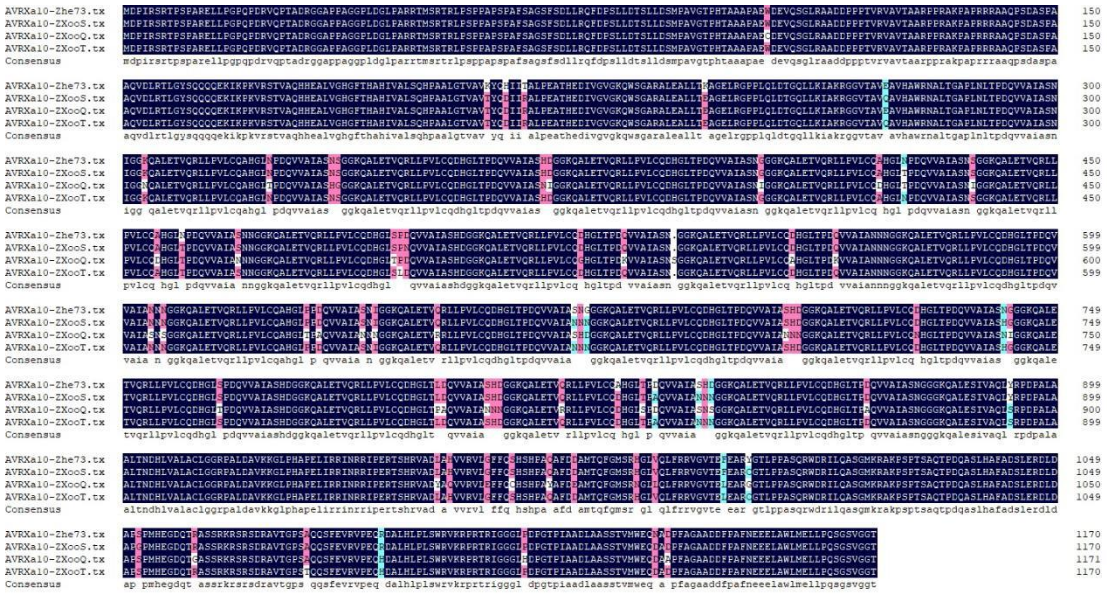

Figure S6. Comparison results of the *AvrXa10* genes amino acid sequence in Zhe173, ZXooS, ZXooQ, and ZXooT strains.

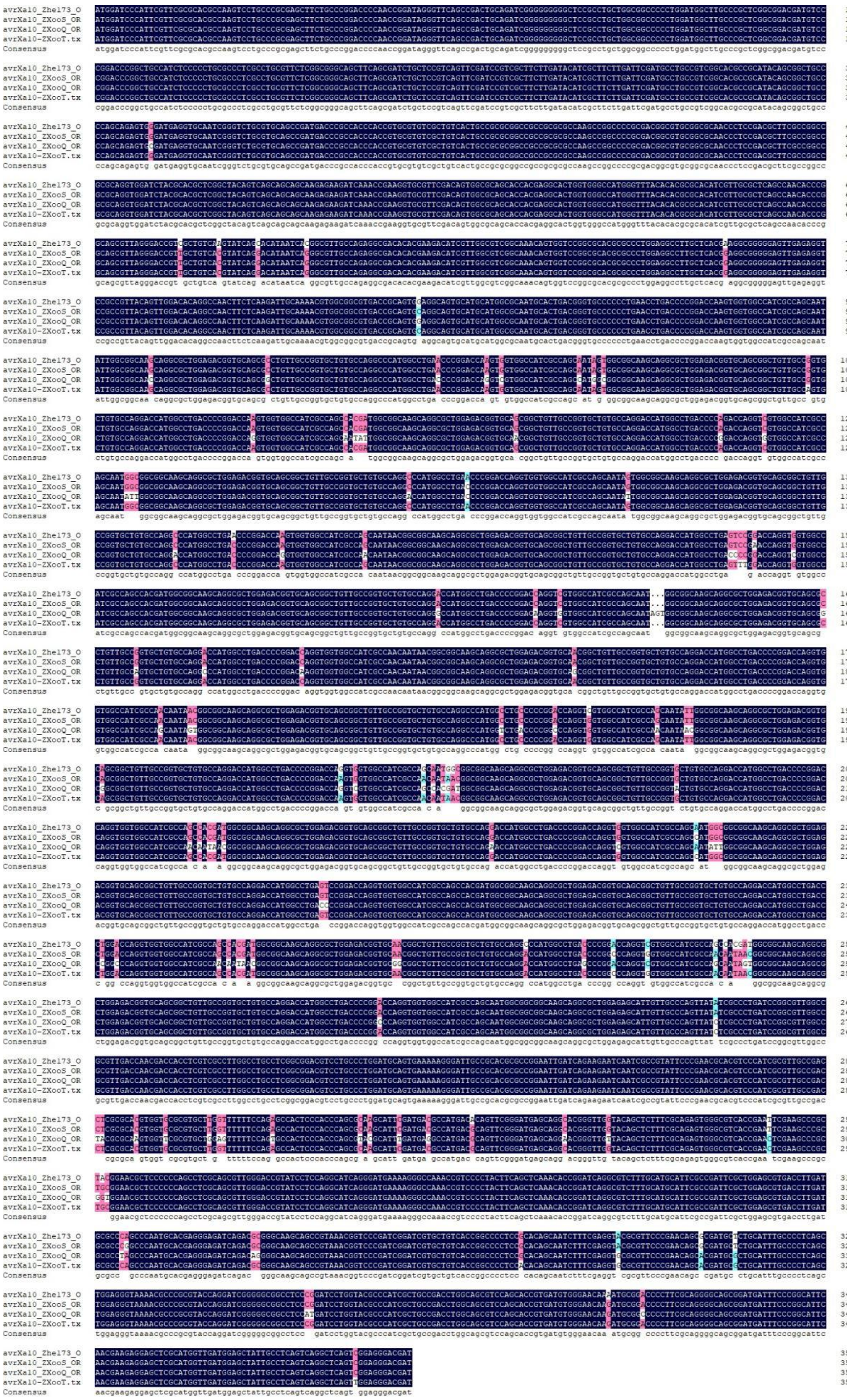

Figure S7. Comparison results of the *AvrXa10* genes nucleotide sequence in Zhe173, ZXooS, ZXooQ, and ZXooT strains.

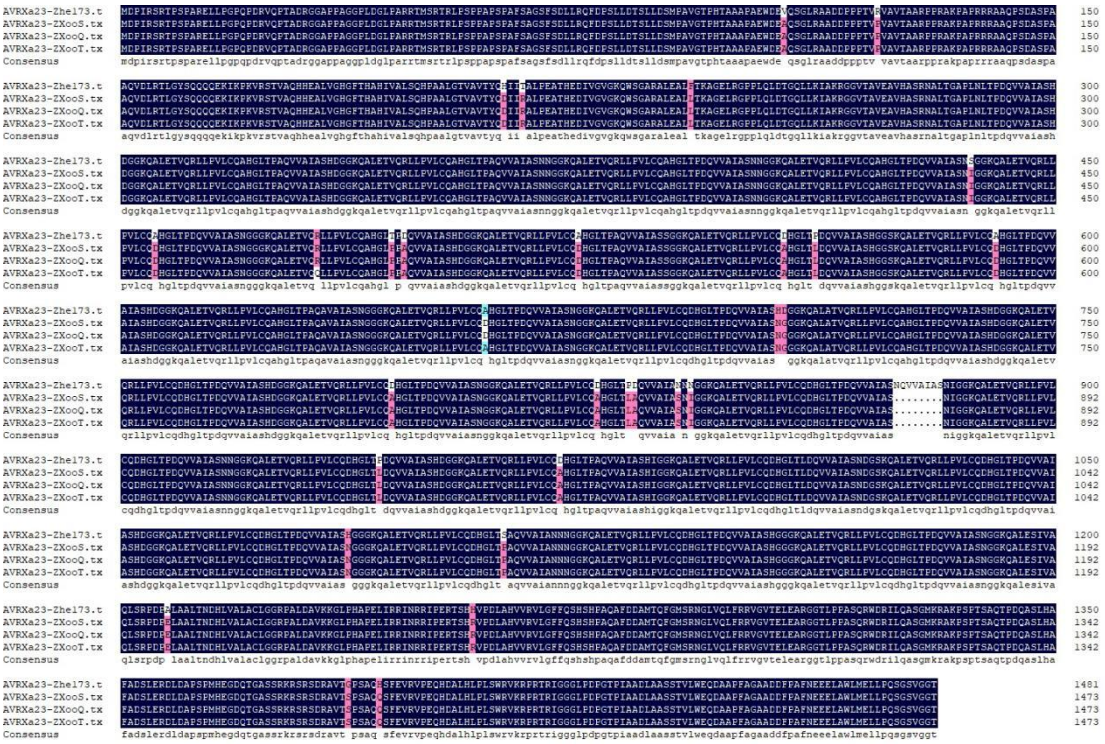

Figure S8. Comparison results of the *AvrXa23* genes amino acid sequence in Zhe173, ZXooS, ZXooQ, and ZXooT strains.

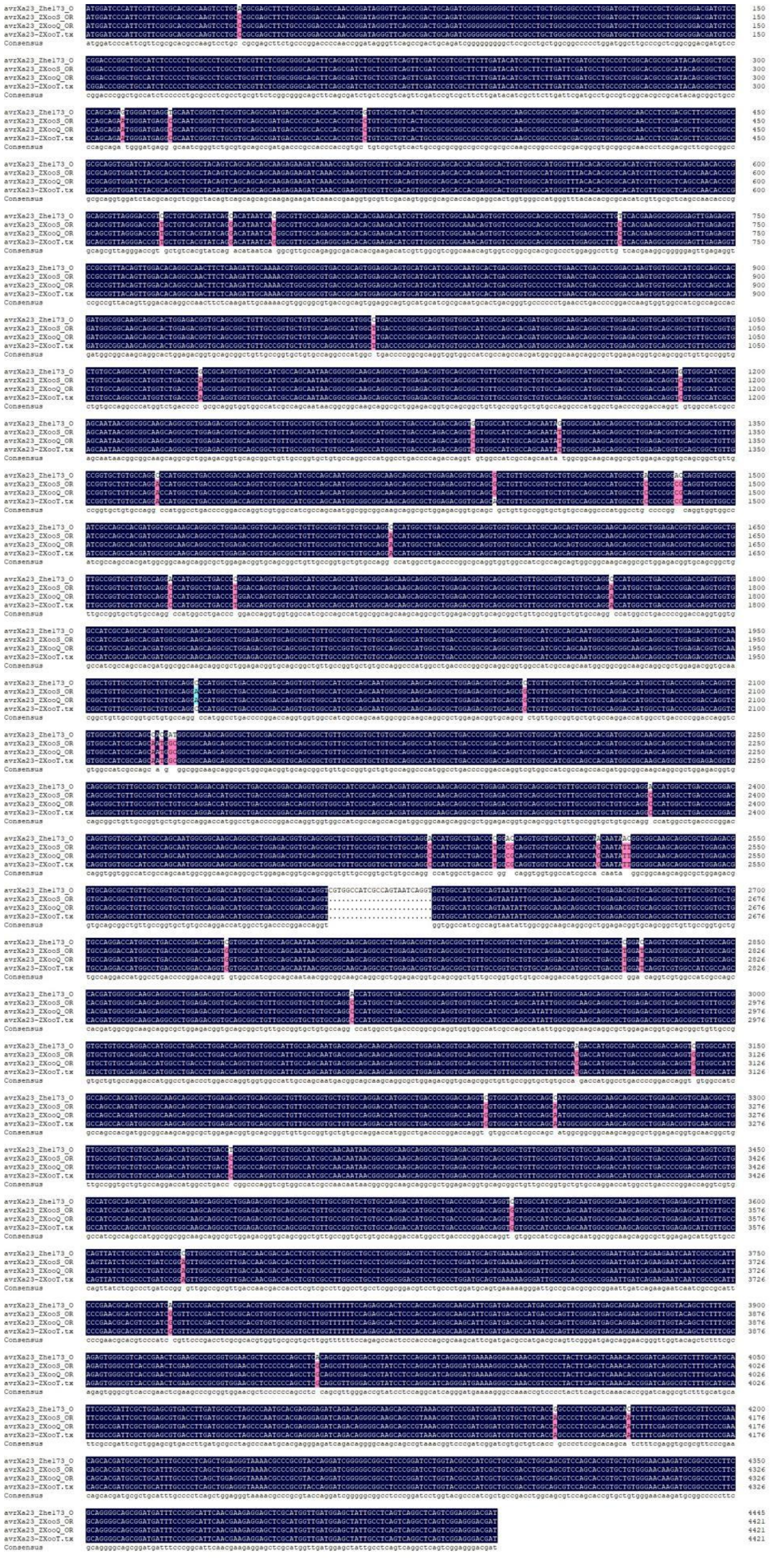

Figure S9. Comparison results of the *AvrXa23* genes nucleotide sequence in Zhe173, ZXooS, ZXooQ, and ZXooT strains.

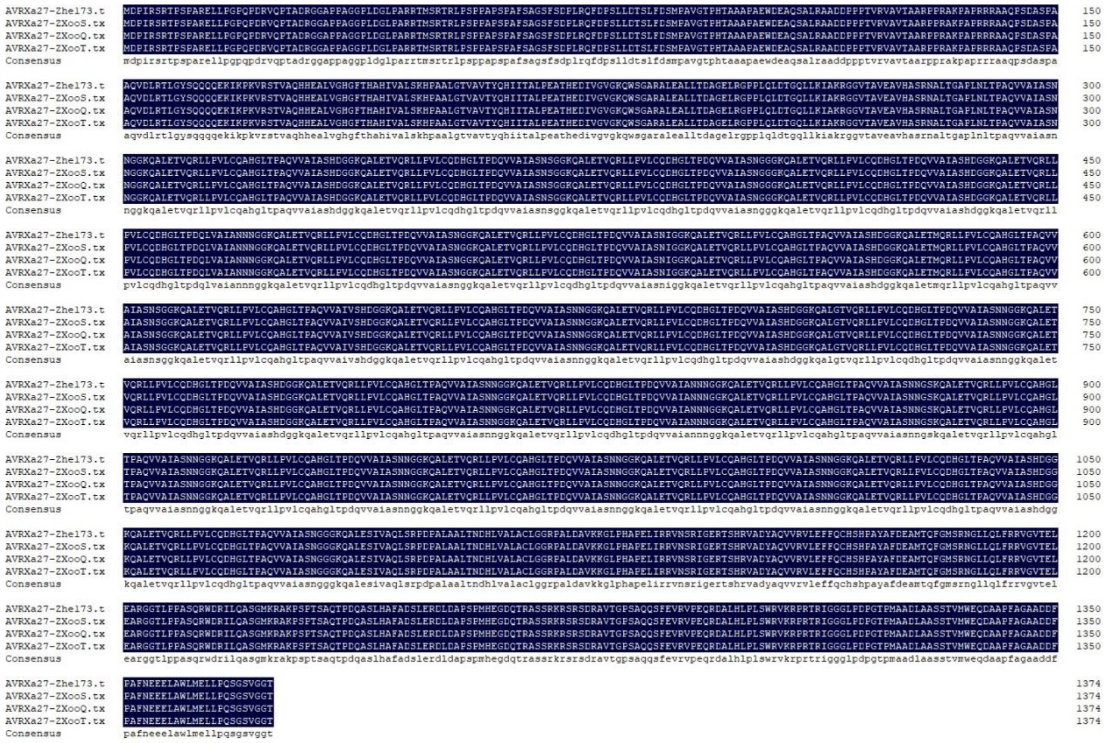

Figure S10. Comparison results of the *AvrXa27* genes amino acid sequence in Zhe173, ZXooS, ZXooQ, and ZXooT strains.

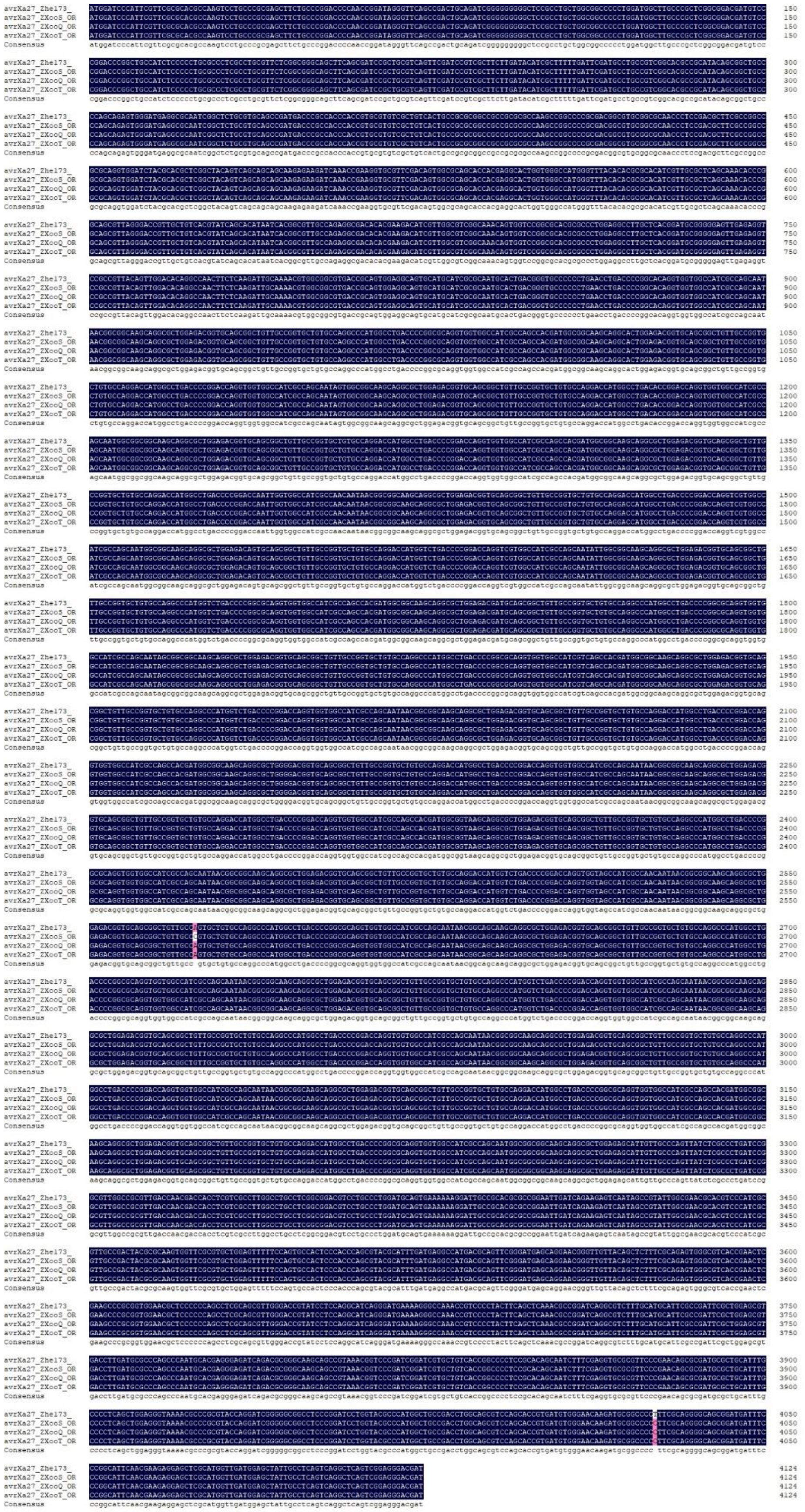

Figure S11. Comparison results of the *AvrXa27* genes nucleotide sequence in Zhe173, ZXooS, ZXooQ, and ZXooT strains.

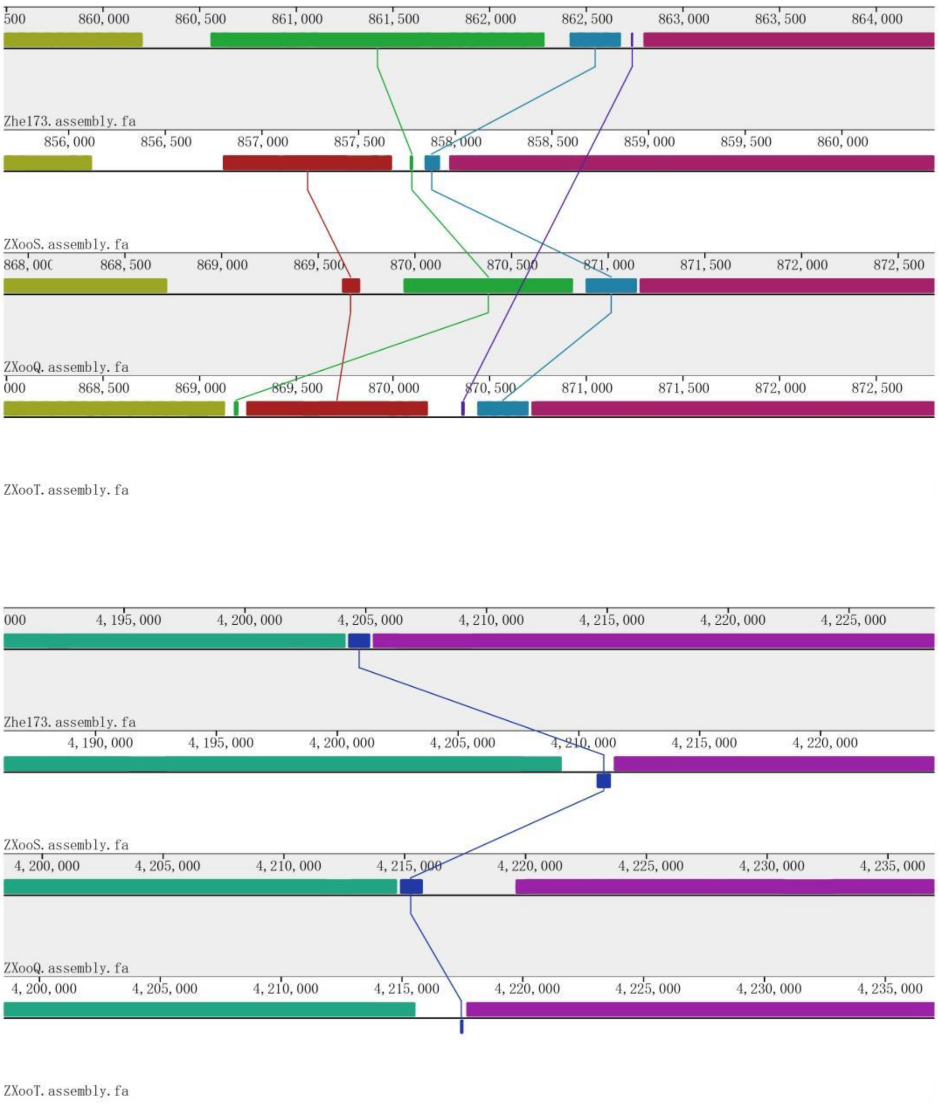

Figure S12. Comparison of whole genome between Zhe173 and novel strains.
